# Decoding Movement Goals from the Fronto-Parietal Reach Network

**DOI:** 10.3389/fnhum.2017.00084

**Published:** 2017-02-24

**Authors:** Hanna Gertz, Angelika Lingnau, Katja Fiehler

**Affiliations:** ^1^Experimental Psychology, Justus-Liebig University GiessenGiessen, Germany; ^2^Department of Psychology, Royal Holloway University of LondonEgham, UK; ^3^Center for Mind/Brain Sciences, University of TrentoMattarello, Italy

**Keywords:** reach planning, sensorimotor integration, fMRI, MVPA, ambiguous reach goals

## Abstract

During reach planning, fronto-parietal brain areas need to transform sensory information into a motor code. It is debated whether these areas maintain a sensory representation of the visual cue or a motor representation of the upcoming movement goal. Here, we present results from a delayed pro-/anti-reach task which allowed for dissociating the position of the visual cue from the reach goal. In this task, the visual cue was combined with a context rule (pro vs. anti) to infer the movement goal. Different levels of movement goal specification during the delay were obtained by presenting the context rule either before the delay together with the visual cue (specified movement goal) or after the delay (underspecified movement goal). By applying functional magnetic resonance imaging (fMRI) multivoxel pattern analysis (MVPA), we demonstrate movement goal encoding in the left dorsal premotor cortex (PMd) and bilateral superior parietal lobule (SPL) when the reach goal is specified. This suggests that fronto-parietal reach regions (PRRs) maintain a prospective motor code during reach planning. When the reach goal is underspecified, only area PMd but not SPL represents the visual cue position indicating an incomplete state of sensorimotor integration. Moreover, this result suggests a potential role of PMd in movement goal selection.

## Introduction

It is debated whether the posterior parietal cortex (PPC) maintains retrospective visuospatial representations (Gottlieb and Goldberg, 1999; Bisley and Goldberg, 2003) or prospective motor representations of upcoming movement goals (for a review, see Andersen and Buneo, 2002). There has been a vast amount of work showing that PPC is a core area for planning and guiding reaching movements in both monkeys (Snyder et al., 1997; Batista and Andersen, 2001; Gail and Andersen, 2006) and humans (Connolly et al., 2003; Culham and Valyear, 2006). Previous research in humans has found that subregions of the PPC represent the movement effector (Connolly et al., 2003; Medendorp et al., 2005; Beurze et al., 2007, 2009; Gallivan et al., 2011a; Heed et al., 2011; Leoné et al., 2014), the orientation of hand/wrist (Monaco et al., 2011; Barany et al., 2014), the grip and transport component (Cavina-Pratesi et al., 2010), the availability of visual information (Filimon et al., 2009), the reachability of a target object (Gallivan et al., 2009), and the type of motor act (Fabbri et al., 2010, 2014; Gallivan et al., 2011b, 2013).

One key aspect of reach planning and execution is the spatial representation of the movement goal. Movement direction selectivity during reach execution has been demonstrated in human PPC, in particular in the superior parietal lobule (SPL) and intraparietal sulcus (IPS), as well as in the dorsal premotor cortex (PMd; Fabbri et al., 2010, 2014; Haar et al., 2015). Likewise, during reach planning SPL and IPS encode the position of the movement goal to be acted upon (Beurze et al., 2007, 2009; Gallivan et al., 2011a). In these studies, however, the visual cue spatially corresponded with the movement goal leaving open whether PPC and PMd rely on a retrospective sensory code or a prospective motor code. The PPC as well as the PMd have been further associated with sensorimotor integration showing higher activation when information about both the effector and the movement goal is given than when only one piece of information is available (Beurze et al., 2007, 2009; Heed et al., 2011; Bernier et al., 2012). It remains unclear how situations with ambiguous movement goals are represented in reach-related areas.

In a recent functional magnetic resonance imaging (fMRI) study, we applied a pro-/anti-reach task and showed that during reach planning the visual movement goal rather than the visual cue is represented in the SPL contralateral to the moving effector (Gertz and Fiehler, 2015). Moreover, we presented a context rule cue (pro vs. anti) before (specified movement goal) or after (underspecified movement goal) a delay and found that underspecified movement goals, compared to specified movement goals, yield weaker activation that is restricted to the parietal cortex (Gertz and Fiehler, 2015). In the current study, we present a re-analysis of the same data reported in Gertz and Fiehler (2015) using multi-voxel pattern analysis (MVPA). It has been demonstrated that MVPA can detect more subtle and fine-grained characteristics of spatial encoding processes (Gallivan et al., 2011a,b; Fabbri et al., 2014; Haar et al., 2015) and thus offers a complementary and more in depth investigation compared to univariate fMRI analyses. It allows us to directly compare our previous results of the univariate analyses with the new results based on multivariate analyses and to identify commonalities and differences in the results.

While earlier studies assumed one core PPC region for reaching, the putative human homolog of monkey parietal reach region (PRR; Connolly et al., 2003), more recent studies argue for multiple reach-related areas within PPC possibly following a functional gradient with different weightings from anterior to posterior areas, e.g., of effector and visuospatial information (Beurze et al., 2009; Leoné et al., 2014) or of different sensory input modalities (Filimon et al., 2009). A broad anatomical distinction can be made between an anterior and a posterior cluster within the PPC. A posterior cluster comprises the posterior precuneus (PCu) and posterior IPS (Prado et al., 2005; Filimon et al., 2009). This cluster often extends into the superior parieto-occipital cortex (SPOC; Culham et al., 2008; Gallivan et al., 2011a) located just anterior or posterior to the parieto-occipital sulcus (POS) and is discussed as the human homolog of monkey area V6A (Fattori et al., 2005, 2010). An anterior cluster covers the anterior precuneus (aPCu), sometimes extending into the middle portions of medial IPS (Prado et al., 2005; Filimon et al., 2009; Gallivan et al., 2011b; Bernier et al., 2012). Activation during reach planning may also comprise both the anterior and posterior parts of the SPL (Beurze et al., 2007, 2009; Filimon et al., 2009; Gertz and Fiehler, 2015). Here we used MVPA to re-analyze a data set which was previously analyzed with univariate methods (Gertz and Fiehler, 2015). We investigated whether fronto-parietal regions represent the visual cue or the movement goal, and whether they can distinguish between different levels of movement goal specification. Specifically, we examined different areas in the anterior and posterior PPC, namely anterior portions of Brodmann area 7 in the SPL (SPL 7A) posterior portions of Brodmann area 7 in the SPL (SPL 7P) and anterior IPS (aIPS) and PMd.

## Materials and Methods

### Participants

Nineteen participants (age range 20–29 years; 11 females) were considered for final analyses in this study. All participants were right-handed as assessed with the Edinburgh Handedness Inventory (Oldfield, 1971), had normal vision, and no history of neurological or psychiatric disorders or chronic diseases. They were financially compensated or received course credit for their participation. All participants gave informed written consent according to the Declaration of Helsinki (2008) before the experiment in accordance with the study procedure approved by the local ethics committee of the Justus-Liebig-University Giessen, Germany. For further information about the sample see Gertz and Fiehler (2015).

### Materials and Set-Up

Light-emitting diodes (LEDs) served as visual cues, rule cues and fixation point. To enable a direct view of the LEDs, participants were positioned in the scanner with their head tilted with wedges (~20–30°) inside the head coil. A green LED indicated that participants had to perform a reach towards the remembered position of the visual cue (pro reach), whereas a red LED required moving towards the position mirrored to the centrally located fixation point (anti reach), e.g., to the lower left in case of a visual cue presented at the lower right.

**Figure 1:**
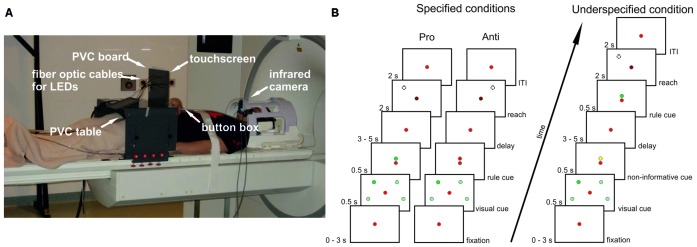
**Setup and experimental design. (A)** Participants lay in the scanner with their head tilted and their index finger on a button box. Right arm reaches were performed to a touchscreen mounted in front of a PVC board. Also attached to this board were optic fiber cables connected to stimuli light-emitting diodes (LEDs) in the control room. The board was mounted to a PVC table placed over the participants’ hips. Eye movements were recorded with an infrared camera. **(B)** Delayed pro-/anti-reach task with different precueing conditions. Context rules (pro, anti) had to be applied to one (single reach trial) or two (double reach trial) visual cues at four possible positions to infer the movement goal. All possible cue positions are illustrated here (light green spheres), but were not visible during the experiment. In this exemplary single-reach trial only one visual cue was presented (dark green sphere). A red fixation LED was visible at the center of the screen throughout the whole trial. In the specified pro condition (left timeline), the context rule was indicated centrally by a green LED above the fixation LED, and reaches were performed toward the position of the previously presented visual cue after a variable memory delay (broken line circle) after the go-cue (change of brightness of the central fixation LED). In the specified anti condition (center timeline), the context rule was indicated by a red LED above the fixation LED. Reaches were performed to the mirror-imaged position of the visual cue (broken line circle). Different precueing conditions were introduced to vary the information available during the memory delay. In the specified pro and anti conditions, both the visual cues and the context rule were available before the delay. In the underspecified conditions (right timeline), only the visual cue was available during the memory delay, whereas the context rule was given immediately after the delay prompting participants to start the respective reaching movement. An additional task-irrelevant yellow cue was presented above the fixation LED before the delay to keep visual input constant. The timeline for underspecified conditions shows an exemplary pro trial, with a green LED above the fixation LED presented after the delay.

An MR-compatible 10.4″ touch screen panel (Magic Touch, Keytec, Inc., Garland, TX, USA) was used to record reaching endpoints. Before and after movement execution participants continuously pressed a button of a custom-made MR-compatible button box placed on their abdomen with their right index finger. For further information about the set-up see Figure 1A and Gertz and Fiehler (2015).

### Task

We adapted a delayed reach task with different cueing conditions from an electrophysiological study in monkeys (Westendorff et al., 2010; Figure 1B). This task allowed us to separate the position of the visual cue from the position of the movement goal by introducing a context rule (pro vs. anti) that had to be applied to one (single reach trial) or two (double reach trial) visual cues. By applying the context rule either before (specified condition) or after the delay (underspecified condition), we were able to manipulate the amount of information available during the delay period resulting in conditions with specified or underspecified movement goals.

In the specified condition (Figure 1B, left panel), the visual cue and the rule cue were presented consecutively. Thus, all information required for setting up a movement plan was available during the following delay period. As soon as the central fixation LED was dimmed (= go-cue) participants started right arm reaches to the remembered visual cue position. In the underspecified condition (Figure 1B, right panel) the visual cue and an additional non-informative cue were presented before the delay. Thus, during the delay period participants knew the position of the visual cue but were uninformed about the reach goal (pro- vs. anti-reach). The rule cue was presented after the delay, followed by the go-cue indicating to start the reach.

In addition to the randomized trial structure with jittered delay durations we varied the number of reaches. Participants performed 50% single-reach trials and 50% double-reach trials. We did so to ensure that planning-related activation is not reduced due to predictability of the target position which may result in stereotyped movements (see, Dassonville et al., 1998; Berndt et al., 2002). In single-reach trials, participants reached to one of four possible visual cue positions, two located in the left and two in the right hemifield (Figure 1B). In double-reach trials, two visual cues were presented successively without a delay, i.e., the second cue was presented right after the first cue was extinguished. Double reaches were performed from the start position to the 1st visual cue position and from there to the 2nd visual cue position (pro reach trial) or from the start position to the mirrored positions of the 1st and 2nd visual cues (anti reach trials) following the order of the visual cue presentation. Both reach goals always fell into the same visual hemifield so that all reaches were either performed within the left or right visual field. Contrasting single- and double-reach trials did not reveal significant differences in the BOLD response. To confirm this finding with more sensitive methods, we used MVPA to decode the number of movement goals from the activation patterns in our ROIs. Preprocessing and MVPA procedures were carried out as described for all subsequent analyses in Sections “Preprocessing” and “MVPA”. In the underspecified condition, participants may plan both possible movements in single reach trials, and all four possible movements in double reach trials. Due to this uncertainty we only used parameter estimates (PEs) from the specified conditions for classification. We trained and tested the classifier on single reach trials (specified pro single, specified anti single) and double reach trials (specified pro double, specified anti double). ROIs were defined as described in Section “ROI Definition”. In none of the ROIs the decoding accuracy was significantly above chance (left PMd: 0.508, uncorrected *p* = 0.29; left anterior SPL: 0.524, uncorrected *p* = 0.08; right anterior SPL: 0.48, uncorrected *p* = 0.88; left posterior SPL: 0.518, uncorrected *p* = 0.09; right posterior SPL: 0.496, uncorrected *p* = 0.59; left aIPS: 0.5, uncorrected *p* = 0.5). The results indicate that even with more sensitive analyses the number of movement goals cannot be distinguished in our ROIs and confirm our findings from univariate analyses. We therefore collapsed single- and double-reach trials for all further analyses.

### Design of the fMRI Experiment

We applied a rapid event-related design. Trials of the specified and underspecified conditions were presented interleaved in random order. Each condition (specified pro, specified anti, underspecified) was repeated 64 times, resulting in 192 trials and a total duration of about 35 min. For further information about the design of the fMRI experiment see Gertz and Fiehler (2015).

### Behavioral Analyses

We assessed individual reach endpoint errors and analyzed the rate of correct responses. We also analyzed the time elapsed from the onset of the go-cue until the first touch, termed as reaction time + movement time (RT + MT). For further information about the behavioral analyses see Gertz and Fiehler (2015).

### Imaging Parameters

The imaging parameters are identical to those reported in Gertz and Fiehler (2015).

### Preprocessing

Imaging data were preprocessed using the fMRI of the brain (FMRIB) Software Library (FSL; version 5.0.2^1^). Preprocessing included the following steps: (1) realignment and motion correction using FSL’s motion correction tool MCFLIRT (Jenkinson et al., 2002); (2) EPI outlier volume detection (fMRI artifact correction tool; Bertram Walter, Bender Institute of Neuroimaging, Giessen, Germany); (3) non-brain tissue removal (FSL’s brain extraction tool BET; Smith, 2002); (4) B_0_-unwarping using fieldmaps; (5) temporal high-pass filtering with a cutoff of 144 s; (6) slice timing correction; and (7) registration of individual functional images to structural images, as well as non-linear registration of individual structural images to the Montreal Neurological Institute (MNI) space (FMRIB’s Non-linear Image Registration Tool; Smith et al., 2004; Andersson et al., 2010). For further information about the preprocessing of the fMRI data see Gertz and Fiehler (2015).

In the following, we set up separate general linear model (GLM) analyses for ROI definition and extraction of PEs for MVPA of the six experimental conditions, resulting from a combination of task (pro, anti, underspecified) and position of the visual cue (left, right). To identify group level peaks for ROI definition, we applied a Gaussian kernel of 5 mm full-width-half-maximum (FWHM) for spatial smoothing. To extract the PEs for MVPA on individual data, data were spatially smoothed with a smaller Gaussian kernel of 2 mm FWHM. Other than that, preprocessing was identical for the two analyses.

### ROI Definition

ROIs were defined on the basis on individual univariate statistical contrasts (PRO + ANTI + UNDERSPECIFIED) > FIX, combined with anatomical masks from the Juelich anatomical atlas (Eickhoff et al., 2007). Importantly, this procedure does not introduce any bias towards one of the experimental conditions (PRO, ANTI, UNDERSPECIFIED) and thus prevents circular analysis (Kriegeskorte et al., 2009; for similar approaches, see e.g., Ariani et al., 2015; Filimon et al., 2015; Wurm et al., 2016).

**Figure 2:**
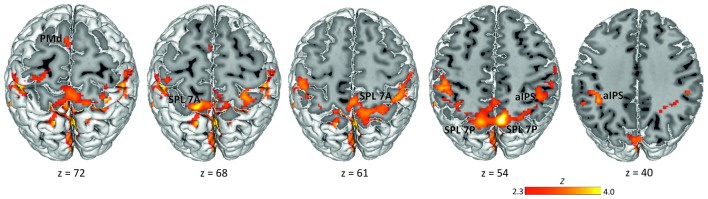
**Delay period activation across conditions.** Activation maps were obtained by calculating one baseline contrast across the three experimental delay conditions (PRO + ANTI + UNDERSPECIFIED) > FIX (*Z* > 2.3, corrected cluster probability threshold *p* = 0.05; *N* = 19). Labels indicate the location of activation peaks used for ROI definition. PMd, dorsal premotor cortex; SPL 7A, anterior portions of Brodmann area 7 in the superior parietal lobule; SPL 7P, posterior portions of Brodmann area 7 in the superior parietal lobule; aIPS, anterior intraparietal sulcus.

Data analysis was performed using the GLM implemented in FSL’s FMRI Expert Analysis Tool FEAT v6.00 (Smith et al., 2004; Jenkinson et al., 2012). FMRIB’s improved linear model (FILM) was used to estimate voxel-wise time series autocorrelation for prewhitening of the time series and thereby improve efficiency of the model. We defined the delay phase (3–5 s from the offset of the rule cue in specified conditions and of the non-informative cue in the underspecified condition) as the period of interest for putative movement planning. We modeled one separate delay predictor for each experimental condition (specified conditions pro and anti, underspecified condition): PRO, ANTI, UNDERSPECIFIED. Note that here we collapsed data across visual cue positions (left, right). In addition to these delay predictors, we defined the fixation interval (FIX), the presentation of the spatial cue, the presentation of the rule cue, and the movement period as predictors of no interest. Each predictor was defined as a boxcar function with the magnitude of 1. Predictors were convolved with a double-Gamma hemodynamic response function in order to model the late undershoot. We also added the temporal derivative to our model to achieve a better fit to the data (Friston et al., 1998). Figure 2 displaying the delay activation overlaid on the MNI 152 template MNI-Colin27 brain template (MNI, Montréal, Canada; Holmes et al., 1998) was created using the Multi-image Analysis GUI (Mango, Research Imaging Institute, San Antonio, TX, USA).

To define the ROIs, we first calculated one baseline contrast across the three experimental delay conditions: (PRO + ANTI + UNDERSPECIFIED) > FIX. For individual analyses, *Z* statistic images were thresholded at *p* < 0.05, corrected for multiple comparisons using Gaussian random field theory (GRF; Worsley et al., 1996). For group-level analyses, PEs were assessed with a mixed effects model, with the random effects component of variance estimated using FSL’s FLAME stage 1 procedure (Beckmann et al., 2003; Woolrich et al., 2004). *Z* (Gaussianized T) statistic images were generated using a *Z* statistics threshold of 2.3 and a corrected cluster probability threshold of *p* = 0.05 using GRF (Worsley et al., 1996). Subsequently, we used the Juelich probabilistic cytoarchitectonic atlas (Eickhoff et al., 2007) to identify regions exhibiting a signal peak in the group level analysis. To ensure that the defined ROIs were anatomically precisely located, we multiplied the activations of the group level baseline contrast with an anatomical mask of each (sub-) region. We applied anatomical masks of the Juelich atlas (Eickhoff et al., 2007) which are based on histological processing and cytoarchitectonic analyses of 10 postmortem human brains. The resulting cytoarchitectural areas are probability maps. For ROI definition, we included all voxels that had a probability of at least 50% as being part of the respective anatomical region. The resulting group-activation-bound anatomical masks in standard MNI space were transformed to individual functional space for each participant separately using FSL’s applywarp. In a next step, we detected the individual signal peaks within the activation-bound anatomical masks using FSL featquery, and placed a sphere with a radius of 10 mm around the corresponding coordinate. We did so to also account for individual activation patterns. Finally, we masked the individual spheres with the original anatomical Juelich masks (again transformed to individual functional space) to ensure that the individual ROIs only comprised voxels of the respective regions. ROIs comprised at least 10 voxels with a voxel size of 3 × 3 × 4 mm (for the mean size of the ROIs see Table 1). Note that we therefore excluded the right aIPS (4.7 voxels) from further analyses.

**Table 1:** **Results of ROI multivoxel pattern analysis (MVPA) and *t* tests against chance for visual cue and movement goal decoding**.

		Mean size (voxels)	Visual cue				Movement goal			
			Accuracy	SEM	*t*	*p*	Accuracy	SEM	*t*	*p*
SPL 7A	Left	45.8	0.543	0.016	2.60	0.009^⋄^	0.541	0.019	2.13	0.023*
	Right	39.9	0.505	0.013	0.38	0.354	0.549	0.014	3.43	0.002^⋄^
SPL 7P	Left	29.1	0.508	0.015	0.56	0.291	0.533	0.018	1.8	0.044*
	Right	41.3	0.487	0.017	−0.77	0.774	0.553	0.023	2.25	0.019*
aIPS	Left	19.6	0.480	0.017	−1.17	0.873	0.487	0.017	−0.79	0.781
PMd	Left	37.3	0.536	0.021	1.69	0.054	0.544	0.022	2.0	0.030*

^⋄^ Significant p values (FDR corrected for number of tests × number of ROIs). *Significant p values (uncorrected).

### MVPA

We used MVPA to examine if and how reach-related areas functionally differ in encoding visual cue or movement goal positions, and movement goals at different levels of specification during the delay period of a pro-/anti-reach task. To do so, we first computed PEs for six experimental conditions (pro, anti, underspecified combined with the visual cue position left vs. right).

As we applied a rapid-event related design with interleaved trial structure we artificially split up the functional scan into eight runs. To avoid temporal dependencies between the runs we randomized all trials of each of the six conditions (32 per condition) and combined four trials to one predictor per condition for each of the eight runs. Thus, the six predictors of interest per run were: PRO_LEFT, PRO_RIGHT, ANTI_LEFT, ANTI_RIGHT, UNDERSPECIFIED_LEFT, and UNDERSPECIFIED_RIGHT (LEFT and RIGHT refer to the position of the visual cue). Predictors were defined with the onset of the delay period for a fixed duration of 3 s and a magnitude of 1. In addition, we modeled the fixation period (FIX), the visual cue presentation, the rule cue presentation, and the reach execution as predictors of no interest as described before (see “ROI Definition” Section). In the following, we set up one GLM for each run and participant in FEAT (Smith et al., 2004; Jenkinson et al., 2012) including the FILM prewhitening procedure and contrasted the predictor of each condition to the fixation period, resulting in six contrasts: PRO_LEFT > FIX, PRO_RIGHT > FIX, ANTI_LEFT > FIX, ANTI_RIGHT > FIX, UNDERSPECIFIED_LEFT > FIX, UNDERSPECIFIED_RIGHT > FIX. We thus obtained 48 PEs for the delay period per participant (6 conditions × 8 runs) used for MVPA.

MVPA was performed using a linear-discriminant analysis (LDA)-based classifier as implemented in the CoSMoMVPA toolbox (Oosterhof et al., 2016). The following steps were performed for every participant and ROI separately. Classification accuracies were computed using leave-one-run-out cross-validation, so that the classifier was trained using seven runs and tested on the remaining pattern of one run. For each participant this procedure was repeated seven times each time leaving out another run as a test pattern. The resulting classification accuracies were averaged per test.

Using MVPA, we pursued two main goals. First, we examined whether reach-related areas encode the spatial position of the visual cue or the (inferred) movement goal, i.e., the combination of visual cue and context rule, during the delay period of the specified conditions. To decode the visual cue position, we trained and tested the classifier on the conditions pro left and anti left vs. the conditions pro right and anti right. To decode the movement goal position we trained and tested the classifier on planned movements to the left (pro left, anti right) vs. movements to the right (pro right, anti left).

Second, we aimed to decode the level of movement goal specification (specified vs. underspecified) and thereby identifying regions potentially involved in sensorimotor integration. The classifier was trained on conditions with underspecified movement goals (underspecified left, underspecified right) vs. conditions with specified movement goals (pro left, pro right, anti left, anti right). To account for the different number of specified (4) and underspecified conditions (2), we balanced the number of samples per class by randomly choosing two out of the four specified conditions in each run of the training set.

In addition, we performed two exploratory analyses. First, we aimed to decode the type of movement goal in order to test for differences in the neural representation of directly cued vs. inferred movement goals as it has been found in monkey; for instance, a preference for stimulus-based representation of directly cued goals in monkey PRR, and for inferred movement goals in monkey PMd (Gail et al., 2009). We therefore trained the classifier on the conditions pro left and pro right (cued movement goals) vs. anti left and anti right (inferred movement goals). Next, we tested which ROIs encode the position of the visual cue despite underspecified movement goals to investigate whether the same regions representing specified reach goals likewise represent underspecified reach goals. To do so, we separately trained the classifier on the conditions underspecified left vs. underspecified right.

We computed a one-tailed one-sample *t* test per ROI against the theoretical chance level of 50% in order to assess statistical significance. Statistical results were FDR corrected for the number of one-sample *t* tests (6 ROIs × 5 tests; Benjamini and Hochberg, 1995).

To determine whether a region is specialized to encode the visual cue or the movement goal position in specified conditions we ran a two-sample *t* test per ROI testing the accuracy of the visual cue against the accuracy of the movement goal. If a region is specialized for encoding the visual cue position, it should exhibit a decoding accuracy significantly above chance level for the visual cue position, but a non-significant decoding accuracy for the movement goal position as assessed by the *t* tests. In addition, it should also show a significantly higher decoding accuracy for the visual cue position than for the movement goal position. However, if a region is specialized for movement goal encoding decoding accuracy should be significantly above chance for the movement goal and not significantly higher than chance for the visual cue. Moreover, one would expect a significantly higher decoding accuracy for the movement goal than for the visual cue.

## Results

### Behavioral Results

As reported in Gertz and Fiehler (2015) there was no significant effect of condition on the percentage of correct responses (*F*_(3,54)_ = 1.954, *p* = 0.146). RT + MT also did not differ between the four conditions (*F*_(3,54)_ = 1.115, *p* = 0.318), specified pro (*M* = 1299 ms, *SD* = 261), specified anti (*M* = 1317 ms, *SD* = 295), underspecified pro (*M* = 1254 ms, *SD* = 483) and underspecified anti (*M* = 1369 ms, *SD* = 519).

### Univariate Results

To define ROIs for the subsequent MVPA, we computed a group baseline contrast for the delay period across all conditions (pro, anti, underspecified). This contrast revealed widespread activation most pronounced in the left and right SPL covering lateral and medial aspects of BA 7 and extending to adjacent left and right aIPS, left and right inferior parietal lobule, and left and right primary somatosensory cortex (Figure 2). We further detected activation in the right frontal pole extending into the orbitofrontal cortex and the parahippocampal gyrus, and in the left frontal pole extending into the left middle and inferior frontal gyrus. Finally, activation was revealed in the dorsal part of the premotor cortex in BA 6.

Previous studies on reach execution identified movement direction encoding in the SPL, adjacent IPS, as well as in PMd (Fabbri et al., 2010, 2014). Therefore, we focused subsequent analyses on these regions. In order to test for differences in the representation of the visual cue and the movement goal in posterior and anterior regions of the PPC (see, Beurze et al., 2007, 2009; Filimon et al., 2009; Heed et al., 2011), we split up the delay-related SPL activation into an anterior and a posterior cluster per hemisphere. To do so, we used the probabilistic histological maps of the Jülich atlas (Eickhoff et al., 2007) which anatomically defines an anterior (7A) and a posterior (7P) portion of the SPL (Scheperjans et al., 2008). While the reach-related posterior PCu, posterior IPS (Prado et al., 2005; Filimon et al., 2009) and SPOC (Culham et al., 2008; Gallivan et al., 2011a) fall into the cluster SPL 7P, the aPCu and medial IPS (Prado et al., 2005; Filimon et al., 2009; Gallivan et al., 2011b; Bernier et al., 2012) fall into the cluster SPL 7A. The PCu activation associated with movement goal encoding we found in our univariate study (Gertz and Fiehler, 2015) covered both SPL 7A and 7P.

Based on the activations of the baseline contrast together with the anatomical maps, we defined ROIs for the two SPL subregions, SPL 7A (peak group MNI coordinates: left −12 −66 68, right 28 −64 64) and SPL 7P (peak group MNI coordinates: left −12 −78 54, right 6 −76 54), adjacent left aIPS (peak group MNI coordinates: −38 −52 40), as well as the left PMd (peak group MNI coordinates: −4 −4 72).

### MVPA Results

We used ROI-based MVPA to examine whether the visual cue and/or the movement goal is encoded in the parieto-frontal reaching network. We focused our analyses on the anterior and posterior SPL, previously discussed as human PRRs, the left aIPS and the left PMd. Second, we aimed to decode different types of movement goals (directly cued vs. inferred). And third, we investigated whether reach-related areas represent the level of movement goal specification (specified vs. underspecified movement goal), and the position of the visual cue in the underspecified conditions.

**Figure 3:**
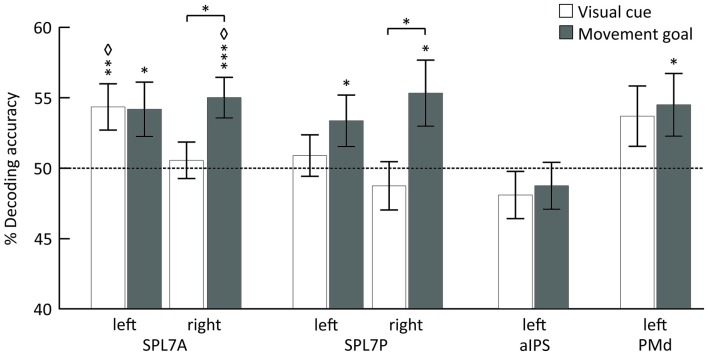
**Mean classification accuracy for decoding the visual cue position (white) and the movement goal (gray).** Error bars indicate SEM, asterisks indicate statistically significant difference from chance (50%) as follows: *p < 0.05; **p < 0.01; ***p < 0.005; ^⋄^FDR corrected for the number of tests. The dotted line represents decoding accuracy at chance (50%). SPL 7A, anterior portions of Brodmann area 7 in the superior parietal lobule; SPL 7P, posterior portions of Brodmann area 7 in the superior parietal lobule; aIPS, anterior intraparietal sulcus; PMd, dorsal premotor cortex.

Using MVPA, we identified different areas encoding the spatial position of the visual cue and the movement goal in the SPL and PMd for combined specified conditions, pro and anti (Figure 3, Table 1).

The position of the visual cue could be decoded in the left SPL 7A and the position of the movement goal in bilateral SPL 7A and 7P and left PMd. In the right SPL 7A and the right SPL 7P, the decoding accuracy was also higher for the movement goal than for the visual cue position (Figure 3, Table 2). In the left aIPS, the decoding accuracy was not above chance for either the visual cue or the movement goal position. Being provided with all necessary information to set up a movement plan biased spatial encoding processes in that network towards the encoding of the respective movement goal.

**Table 2:** **Results of two-tailed *t* tests between visual cue and movement goal**.

		*t*	*p*
SPL 7A	Left	−0.0777	0.939
	Right	2.6197	0.017*
SPL 7P	Left	1.41	0.176
	Right	2.638	0.017*
aIPS	Left	0.236	0.816
PMd	Left	0.2538	0.802

*Significant p values (uncorrected).

None of the ROIs encoded the difference between directly cued and inferred movement goals, i.e., between conditions pro and anti (Table 3).

**Table 3:** **Results of ROI MVPA and *t* tests against chance for decoding specified conditions pro vs. anti**.

		Accuracy	SEM	* t*	*p*
SPL 7A	Left	0.515	0.014	1.06	0.152
	Right	0.5	0.020	0	0.5
SPL7 P	Left	0.518	0.014	1.26	0.113
	Right	0.484	0.019	−0.86	0.801
aIPS	Left	0.512	0.02	0.58	0.283
PMd	Left	0.487	0.02	−0.67	0.743

**Figure 4:**
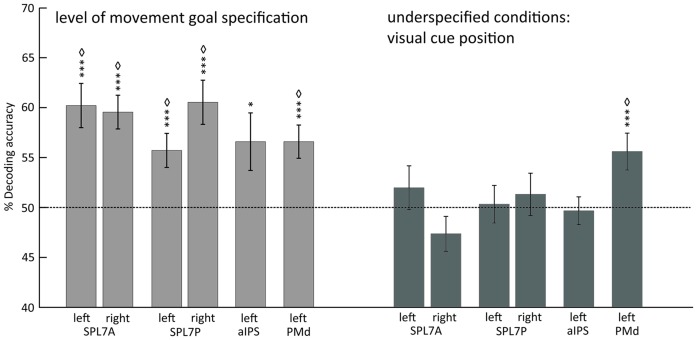
**Mean classification accuracy for decoding the level of movement goal specification (light gray) and the visual cue position in underspecified conditions (dark gray).** Error bars indicate SEM, asterisks indicate statistically significant difference from chance (50%) as follows: *p < 0.05; ***p < 0.005; ^⋄^FDR corrected for the number of tests. Dotted line represents decoding accuracy at chance (50%). SPL 7A, anterior portions of Brodmann area 7 in the superior parietal lobule; SPL 7P, posterior portions of Brodmann area 7 in the superior parietal lobule; aIPS, anterior intraparietal sulcus; PMd, dorsal premotor cortex.

Underspecified vs. specified movement goals could be distinguished in all SPL subregions (left and right SPL 7A, left and right SPL 7P) as well as in left aIPS and left PMd (Figure 4, Table 4). The results demonstrate that different levels of movement goal specification (specified vs. underspecified) but not the type of movement goal (anti-inferred vs. pro-cued) can be distinguished in fronto-parietal reach regions.

**Table 4:** **Results of ROI MVPA and *t* tests against chance for decoding specified vs. underspecified movement goals and visual cue position in underspecified conditions**.

		Level of movement goal specification				Visual cue (underspecified conditions)			
		Accuracy	SEM	*t*	*p*	Accuracy	SEM	*t*	*p*
SPL 7A	Left	0.602	0.022	4.61	0.0001^⋄^	0.52	0.022	0.9	0.19
	Right	0.595	0.017	5.65	0.00001^⋄^	0.473	0.017	−1.51	0.926
SPL 7P	Left	0.557	0.017	3.34	0.0018^⋄^	0.503	0.018	0.175	0.432
	Right	0.605	0.022	4.76	0.000078^⋄^	0.513	0.021	0.62	0.271
aIPS	Left	0.566	0.029	2.28	0.0174*	0.497	0.014	−0.24	0.592
PMd	Left	0.566	0.017	3.95	0.0005^⋄^	0.55	0.018	3.03	0.004^⋄^

^⋄^Significant p values (FDR corrected for number of tests × number of ROIs). *Significant p values (uncorrected).

For underspecified conditions, the position of the visual cue was decoded from the left PMd but not from areas in the PPC (Figure 4, Table 4).

## Discussion

In the present study, we aimed to investigate whether areas of the fronto-parietal reaching network encode the position of the visual cue or the movement goal in a pro-/anti-reach task. Using MVPA we demonstrate that the bilateral SPL and the left PMd encode the position of the movement goal when the movement plan is specified. The right anterior and posterior portions of the SPL (7A and 7P) elicited highest specificity for movement goal encoding. We were able to decode the visual cue position in the left anterior SPL (7A); the same region in which we also decoded the movement goal position. None of the examined areas differentiated between directly cued and inferred movement goals, i.e., between pro- and anti-reach planning. We observed the level of movement goal specification (specified vs. underspecified) to be encoded in all examined ROIs, i.e., bilateral posterior and anterior SPL, left aIPS and left PMd. For conditions with underspecified movement goals, the visual cue position only showed specificity in the left PMd, but not in the PPC. Finally, these novel MVPA results complement our previous findings based on univariate analyses of the same data set (Gertz and Fiehler, 2015).

### Spatial Encoding Processes during Movement Preparation

Our findings from the specified conditions provide evidence that specifying the movement goal biases the encoding in bilateral SPL and PMd towards the position of the upcoming movement goal instead of the visual cue position. The latter seems to be maintained in the left anterior SPL which also encodes the movement goal, showing that the two encoding processes are not necessarily mutually exclusive.

Posterior parietal areas such as SPL and IPS have been suggested to encode the position of the movement goal (Beurze et al., 2007, 2009; Gallivan et al., 2011a). Studies dissociating the positions of the visual target from the movement goal by using reversing prisms (Fernandez-Ruiz et al., 2007) or anti-reaches (Gertz and Fiehler, 2015) reported movement-goal specific activation in SPL. Similarly, single-neuron spiking activity in monkey PRR reflects the position of the movement goal unrelated to visual memory (Kuang et al., 2016). Using MVPA, we found that not only SPL subregions 7A and 7P but also area PMd encode the position of the movement goal. Thus, with MVPA we identified movement goal representations in the PMd which we did not detect using standard univariate analyses of the same data set. Human PMd may thus resemble monkey PMd in that it encodes movement goal positions (Westendorff et al., 2010), and possibly movement directions (Crammond and Kalaska, 1994). Our findings highlight the function of the fronto-parietal network in representing a prospective motor code during movement planning and contribute to the debate about whether areas in the PPC largely maintain visuospatial, sensory codes (Gottlieb and Goldberg, 1999; Bisley and Goldberg, 2003) or whether they are motor-related comparable with frontal motor regions (see, Snyder et al., 1997; Andersen and Buneo, 2002; Andersen and Cui, 2009; Filimon, 2010; Lindner et al., 2010; Filimon et al., 2015).

A preference for reach goal encoding was present in both anterior and posterior portions of the SPL while neither of these areas showed a preference for visual cue encoding. Thus, the present results do not support a functional gradient from posterior to anterior PPC for visual cue and movement goal encoding, respectively, at least in SPL 7 (see, Beurze et al., 2009; Leoné et al., 2014). Nevertheless, the visual cue could be decoded in left SPL 7A, the same area that also encodes the movement goal. This suggests that different neuronal populations within the same area encode the visual cue and the movement goal. The pattern of both visual and motor representations found in the left SPL 7A renders this area as optimal candidate structures for sensorimotor integration.

In area aIPS, MVPA was neither able to decode the position of the visual cue nor the movement goal. Area aIPS is a grasp-selective region showing higher activation during the execution of grasping than reaching movements in monkeys and humans (Murata et al., 2000; Culham et al., 2003) and encoding of grasp vs. reach movement planning as well as of similar grasps on objects with different sizes (Gallivan et al., 2011b). Moreover, aIPS contains overlapping representations of movement direction and grip type and does not show pure directional selectivity (Fabbri et al., 2014) that might hide a representation of the reach goal.

We further demonstrate that none of the examined fronto-parietal regions differentiate the type of movement goal, i.e., directly cued vs. inferred movement goals for pro- and anti-reaches, respectively. This is consistent with the largely overlapping brain activation in the fronto-parietal network we found during planning of pro- and anti-reach movements based on univariate analyses (Gertz and Fiehler, 2015). In monkeys, it has been shown that movement goal tuning in PRR occurs later in anti-reach compared to pro reach trials (Gail and Andersen, 2006). The lack of a differential effect may be due to the fact that decoding was based on a delay period of 3 s, diluting potential effects of response inhibition or movement re-planning in anti-reach trials. In our study, it is likely that participants inferred the movement goal at the very beginning of the delay period so that differences of the type of movement goal were not decodable across the delay. So far, differential activation for pro- and anti-pointing has only been shown in a block-design fMRI study in which more statistical power may have been assigned to obtaining the type of movement goal (Connolly et al., 2000). The fact that we were able to distinguish between movement goals but not between pro- and anti-reaches further emphasizes the importance of the position of the reach goal during reach planning, whereas the way the goal is obtained (directly cued or inferred) seems to be less relevant.

### Hemispheric Asymmetries in the PPC

In the anterior and posterior SPL, we found bilateral representations of specified movement goals, with higher specificity for movement goal encoding in the right SPL, i.e., ipsilateral to the moving effector. Previous univariate studies on spatial encoding processes during reach planning reported movement goal encoding in subregions of the SPL contralateral to the moving effector and thus suggested a contralateral bias in SPL (Medendorp et al., 2005; Fernandez-Ruiz et al., 2007; Gertz and Fiehler, 2015). However, findings from recent MVPA studies likewise argue against strict contralateral effector-specificity during reach planning (Gallivan et al., 2013; Ariani et al., 2015) and execution (Fabbri et al., 2014). During reach execution it has even been shown that right SPL elicits high directional selectivity during both left- and right-hand reaches (Fabbri et al., 2010). This again demonstrates that uni- and multivariate approaches do not necessarily lead to similar results since differences between activation patterns might occur in the absence of amplitude differences of the BOLD response and vice versa (for recent examples, see Leoné et al., 2014; Ariani et al., 2015; Wurm et al., 2016). One may speculate that the movement goal representation in the ipsilateral hemisphere is of importance for the preparation of bimanual actions or of a sudden effector change to left arm reaches.

### Representation of Ambiguous Reach Goals

As we have shown for specified conditions, PPC regions and PMd represent the position of the reach goal. If ambiguous reach goals lead to a parallel specification of multiple reach plans as has been demonstrated in monkeys (e.g., Cisek and Kalaska, 2002, 2005; Klaes et al., 2011), PPC regions and PMd should likewise maintain a spatial representation of the potential reach goals. Here, we found that only area PMd differentiates left from right visual cue positions in underspecified conditions which may represent potential reach goal positions, similar to the results we obtained for the specified conditions. Interestingly, in underspecified conditions PMd showed spatial encoding as revealed using MVPA, but previous univariate analyses revealed a BOLD response not significantly higher than baseline (Gertz and Fiehler, 2015). In specified conditions, on the other hand, PMd likewise encodes spatial positions (of the movement goal), but also exhibits a BOLD response significantly higher than chance. This suggests that spatial encoding processes in PMd in ambiguous conditions are more subtle than when the movement goal is specified, and that MVPA is a suitable tool to examine these processes. The encoding of spatial locations would be in line with the notion that neurons in monkey PMd are tuned to visual cue locations (Hoshi and Tanji, 2006) and are preferably involved in spatial aspects of action, such as active maintenance of visuo-spatial coordinates (Cisek, 2006). Our results indicate that human PMd likewise represents spatial information related to the visual cue when the movement goal is ambiguous. It is important to note that neither our study nor previous fMRI studies can fully disentangle whether PMd encodes both visual cue positions, both movement goal positions, or visual cues and movement goals in parallel. That is, it remains unclear whether PMd represents the visuospatial or the motor component (as predicted by the affordance competition hypothesis; Cisek, 2006) when the reach goal is ambiguous. Monkey PMd represents the behavioral uncertainty about the reach goals, not the uncertainty of the visual information as manipulated by noise added to the visual cue (Dekleva et al., 2016). One may therefore speculate that coactivated populations in PMd maintain potential reach goals at their preferred locations (see, Cisek and Kalaska, 2005) rather than the visual cue. Future research is needed to clarify how “motor” or “visual” the spatial representation of potential reach goals is in area PMd.

When the movement goal is fully specified, PMd is biased towards reach goal encoding. Monkey PMd also engages in goal selection processes based on competition of multiple alternative movement plans (Cisek and Kalaska, 2002, 2005; Cisek, 2006) and seems to be engaged in sensorimotor transformations as it represents both movement goal locations and limb trajectories with a stronger preference for the latter towards movement onset (Shen and Alexander, 1997). Although we cannot address the time course of sensorimotor integration with the current study, one may speculate that the visual cue position is maintained in PMd until the movement goal is specified. Movement goal selection may then happen in PMd before sending this information via feedback projections to the PPC, as has been suggested by electrophysiological studies in monkeys (Pesaran et al., 2008; Westendorff et al., 2010) and fMRI studies in humans (Bernier et al., 2012). Our finding of visual cue encoding in PMd when the movement goal is ambiguous may strengthen the importance of human PMd in reach goal selection.

In contrast to area PMd, we found no evidence for SPL subregions encoding the visual cue position in underspecified conditions, despite the fact that they strongly encode the movement goal position in specified conditions. Movement goal specification seems to be necessary for SPL subregions but not for PMd to elicit spatial representations of reach goals. Using univariate analyses (Gertz and Fiehler, 2015), the posterior SPL elicited activation when confronted with underspecified reach goals but the activation was weaker in comparison to conditions with specified reach goals. Accordingly, here we show that PPC regions and PMd distinguish between different levels of movement goal specification, i.e., delay periods in which the movement goal was specified vs. underspecified. The distinction between specified and underspecified conditions could be a result of mutual inhibition of competing movement plans (see, Cisek, 2006) and/or an incomplete state of sensorimotor integration (see, Beurze et al., 2007; Bernier et al., 2012). Here we show that SPL activation does not represent potential reach goal positions in conditions with ambiguous movement goals in contrast to its role in specified conditions. This is consistent with previous findings of non-spatial preparatory activation in PMd and PPC in conditions in which only the movement goal or the effector to move (Beurze et al., 2007) was known. The role of such non-spatial activation remains widely unclear. Potential explanations have been put forward based on electrophysiological findings in macaques. For example, Snyder et al. (2006) argued that an elevated baseline of non-spatial PRR activity found in underspecified conditions is useful for a rapid development of PRR firing rates that represent the reach goal, once it is specified. The earlier movement goal representation in PRR seems to cause a faster transfer of spatial information to the arm muscles, and thereby lead to shorter RTs. A similar mechanism might account for our findings. An elevated, non-spatial baseline in posterior SPL 7 may facilitate a rapid specification of the reach goal once the context rule (pro or anti) is presented. With these characteristics, posterior SPL 7 may thus be in a “prepare-to-prepare” state rather than in a “prepare-to-move” state as in the specified conditions. A transformation from “prepare-to-prepare” to “prepare-to-move” potentially takes place when the reach goal is selected from the spatial representations in PMd and sent back to PPC as speculated above. Only then the fronto-parietal reaching network might be fully recruited and a spatial representation of reach goals set up in PPC.

Taken together, results from our previous univariate analyses (Gertz and Fiehler, 2015) and the multivariate analyses presented here show that ambiguous reach goals, in comparison to unambiguous (specified) reach goals, yield weaker and non-spatial activation in PPC. By contrast, PMd differentiates between left and right visual cue positions but does not exhibit suprathreshold BOLD responses. Specified and underspecified reach goals thus yield largely disparate cortical representations and suggest that ambiguous reach goals lead to an incomplete state of sensorimotor integration rather than a parallel specification of multiple movement plans.

## Conclusion

We found evidence for movement goal encoding in anterior and posterior regions of the SPL as well as in PMd during reach planning. We conclude that fronto-parietal regions of the reaching network maintain a prospective motor code rather than a retrospective sensory code when the movement goal is specified. Moreover, reach-related fronto-parietal areas can distinguish between different levels of movement goal specification. When confronted with underspecified reach goals, the PMd but not PPC subregions encode the visual cue position which may represent potential reach goals. Our results suggest that situations with ambiguous reach goals result in an incomplete state of sensorimotor integration in the fronto-parietal reach network.

## Author Contributions

HG and KF designed the experiment; HG collected the data; HG and AL analyzed the data; HG, AL and KF wrote the article.

## Conflict of Interest Statement

The authors declare that the research was conducted in the absence of any commercial or financial relationships that could be construed as a potential conflict of interest.

## Footnotes

^1^http://www.fmrib.ox.ac.uk/fsl
